# Magnetostructural transformation and magnetocaloric effect of Sn-bonded Mn_0.66_Fe_0.34_Ni_0.66_Fe_0.34_Si_0.66_Ge_0.34_ composite

**DOI:** 10.1038/s41598-017-18240-x

**Published:** 2018-01-08

**Authors:** Yu Si, Jun Liu, Yuan-yuan Gong, Sheng-yun Yuan, Guo Peng, Gui-zhou Xu, Feng Xu

**Affiliations:** School of Materials Science and Engineering & Herbert Gleiter Institute of Nanoscience, Nanjing University of Science and Technology, Nanjing, 210094 China

## Abstract

Magnetostructural coupling in MnMX (M = Co or Ni, X = Si or Ge) system attracts considerable attention for the accompanied multi-magnetoresponsive effects. However, due to the large stress generated from the structural transformation, the alloys become shattered or powder-like, hindering the further investigation and their applications. The possible solution is to embed the MnMX powders into metal matrix. In this paper, we choose Mn_0.66_Fe_0.34_Ni_0.66_Fe_0.34_Si_0.66_Ge_0.34_ as a representative of MnMX alloy and produce Mn_0.66_Fe_0.34_Ni_0.66_Fe_0.34_Si_0.66_Ge_0.34_/Sn composite bulk by hot pressing. The magnetostructural-coupled composites exhibit an improved rate of the transformation temperature shift by magnetic field and broadened operating temperature range. Additionally, we also propose a simple formula based on the entropy-temperature diagram to calculate the isothermal entropy change, which is consistent with the results obtained by the Maxwell relation.

## Introduction

The alloys which exhibit the coupling between the magnetic transition and structural transformation attract considerable attention due to the abundant magnetostructural-transformation-accompanied magnetoresponsive effects, such as the magnetoresistance, magnetic field-induced-strain, magnetic shape memory and magnetocaloric effects^1–6^. Recently, MnMX-based (M = Co or Ni, X = Si or Ge) alloys are identified as a new system that displays the magnetostructural coupling^7–24^. They experience a martensitic-like magnetostructural transformation between the paramagnetic Ni_2_In-type hexagonal (H) and the ferromagnetic TiNiSi-type orthorhombic (O) phases (H and O phases correspond to Austenite and Martensite, respectively)^7–24^. (In some MnNiGe-based compounds, the transition occurs between the ferromagnetic H and the nonlinear antiferromagnetic O phases^16^). The magnetostructural transformation can be induced not only by the temperature, but also by the magnetic field^7–24^. The magnetic-field-induced structural transformation is accompanied by a considerable magnetic entropy change, suggesting the possible application of the alloys as magnetic cooling refrigerant. However, one characteristic of the H-O transformation in this system is the giant lattice discontinuity^7–24^. As calculated from the reported data^7–24^, the volume expansion (volume(O)/volume(H)-1) is as large as 2–5% (e.g., 2.36% for Mn_0.84_Fe_0.16_NiGe^7^, 3.61% for Mn_0.64_Fe_0.36_NiGe_0.5_Si_0.5_^17^ and 4.24% for (NiMnSi)_0.62_(FeNiGe)_0.38_^18^), and the increase of lattice constant along *c*-axis in H phase (*a*(O)/*c*(H)-1) is higher than 10% (e.g., 12.26% for Mn_0.84_Fe_0.16_NiGe^7^, 12.66% for Mn_0.64_Fe_0.36_NiGe_0.5_Si_0.5_^17^ and 12.79% for (NiMnSi)_0.62_(FeNiGe)_0.38_^18^). The large stress generated from this abrupt transformation turns the prepared samples to be shattered or even powder-like^7,11^, thus prevents the further investigations on them as bulk alloys and makes it infeasible to explore their future applications. Similar phenomenon can also be found in Mn-Fe-P-Si system which exhibits large reversible magnetocaloric effects but poor mechanical integrity^25–27^.

One possible solution is to embed the pulverized samples into a polymer matrix, and thus produce the functional composite^28,29^. This method has been applied in MnCoGe-based and La-Fe-Si-based alloys^28,29^. However, since the thermal conductivity of the polymer matrix is much lower than that of the embedded metallic particles, the heat transfer in the composite is impeded. It apparently affects their application in heat exchangers, such as magnetic cooling refrigerator. To improve the thermal conductivity, researchers have replaced the polymer with metal. For example, in La-Fe-Si-based composites, the metallic glass, low-melting-point metals, Cu and Fe have been employed as the matrix^30–34^. However, the metal-bonding has never been carried out on MnMX systems, which experience the giant lattice discontinuity during the structural transformation. In this work, we select Mn_0.66_Fe_0.34_Ni_0.66_Fe_0.34_Si_0.66_Ge_0.34_ as a representative of MnMX alloy. The reason for us to choose this composition is as following: (i) By introducing Fe, the saturation moment of ferromagnetic O phase is enlarged. Based on this improvement, the magnetization difference (ΔM) between O and H phases is larger than that of other MnMX alloys, which is helpful for the magnetic-field-induced magnetostructural transformation; (ii) the structural transformation is around room temperature (RT)^12^. More information about Mn_0.66_Fe_0.34_Ni_0.66_Fe_0.34_Si_0.66_Ge_0.34_ can be found in ref.^12^. On the other hand, Sn with the melting point of ~232 °C is chosen as the metal matrix for the low-temperature preparation, thus the atomic diffusion can be greatly inhibited with the particles’ properties well maintained^32^. The Mn_0.66_Fe_0.34_Ni_0.66_Fe_0.34_Si_0.66_Ge_0.34_/Sn composites were produced by hot pressing, and their magnetostructural transformation and magnetocaloric effect are reported.

## Results and Discussions

### Structure, morphology and mechanical properties

**Figure 1:**
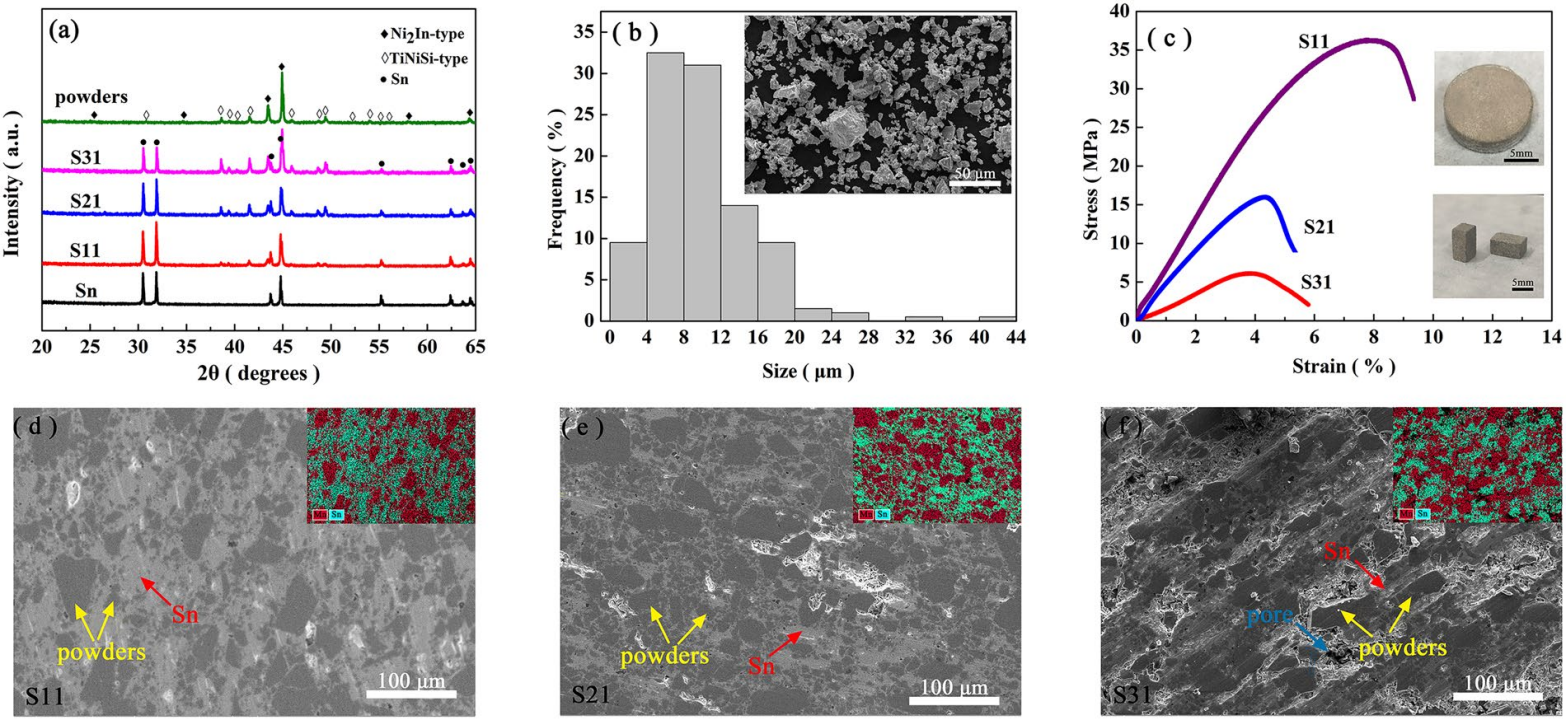
(**a**) XRD patterns for Mn_0.66_Fe_0.34_Ni_0.66_Fe_0.34_Si_0.66_Ge_0.34_ precursors, composites and Sn powders. (**b**) Grain distributions for powders. Inset: SEM image of powders. (**c**) Compressive stress-strain curves for composites. Inset: button-like composite after hot pressing (above) and the processed cuboid (below). (**d**–**f**) SEM micrographs of the surfaces of S11, S21 and S31, respectively. Inset: elemental mapping.

Mn_0.66_Fe_0.34_Ni_0.66_Fe_0.34_Si_0.66_Ge_0.34_ precursor is prepared by the method mentioned in ref.^12^. The room-temperature powder X-ray diffraction (XRD) measurement (Fig. 1a) indicates the coexistence of O and H phases, suggesting that the H-O structural transformation in Mn_0.66_Fe_0.34_Ni_0.66_Fe_0.34_Si_0.66_Ge_0.34_ precursor occurs at around RT. It agrees with the results in ref.^12^ and is also proved by differential scanning calorimetry (DSC) measurement in this work (shown in Fig. S1 in Supplementary information). With the occurrence of H-O structural transformation, the precursor breaks itself into small particles with an average size of ~700 μm. Before synthesizing the Mn_0.66_Fe_0.34_Ni_0.66_Fe_0.34_Si_0.66_Ge_0.34_/Sn composite, these particles were ground into powders. The size of obtained powders ranges from 2 to 30 μm with an average value of 9.94 μm (Fig. 1b). This value was obtained by counting all the powders in the micrograph shown in the inset of Fig. 1b. As the particle size of the precursor is much larger than that of the ground powders, we call the precursor as “bulk” thereafter.

The Mn_0.66_Fe_0.34_Ni_0.66_Fe_0.34_Si_0.66_Ge_0.34_ powders are mixed with the commercial Sn powders with an average size of 43 μm, then hot-pressed into button-like composites. The weight ratios of Mn_0.66_Fe_0.34_Ni_0.66_Fe_0.34_Si_0.66_Ge_0.34_ to Sn were chosen as 1:1, 2:1 and 3:1, respectively. (The corresponding composite samples are named S11, S21 and S31 thereafter). According to the densities of Sn (7.28 g/cm^3^) and Mn_0.66_Fe_0.34_Ni_0.66_Fe_0.34_Si_0.66_Ge_0.34_ (7.17 g/cm^3^. The molecular weight is 156.21 g/mol and the volume of hexagonal unit cell is 72.50 Å^3^), the corresponding volume ratio of Mn_0.66_Fe_0.34_Ni_0.66_Fe_0.34_Si_0.66_Ge_0.34_ to Sn can be calculated, which is 1.02:1, 2.03:1 and 3.05:1 for S11, S21 and S31, respectively. The composites are 13 mm in diameter and 4 mm in height, and can be processed into the cuboid shape with a size of 3.5 mm × 3.7 mm × 7.3 mm by a diamond wire saw (the inset of Fig. 1c). It is notable that if the weight ratio of Mn_0.66_Fe_0.34_Ni_0.66_Fe_0.34_Si_0.66_Ge_0.34_:Sn is higher than 3:1 or the processing is performed without pressure, the composites with special shapes cannot form. The XRD patterns of the composites (Fig. 1a) present the coexistence of the reflection peaks of Sn and Mn_0.66_Fe_0.34_Ni_0.66_Fe_0.34_Si_0.66_Ge_0.34_ with O and H structures. Obvious diffraction patterns from other phases are not found. The micrographs of the composites’ surfaces obtained by scanning electron microscopy (SEM) are shown in Fig. 1d–f. The elemental mapping images (the insets of Fig. 1d–f) show that Mn_0.66_Fe_0.34_Ni_0.66_Fe_0.34_Si_0.66_Ge_0.34_ powders are embedded in Sn matrix. The cracks and holes are not obviously observed in S11 and S21, but appear in S31. They lead to the lowest yield compressive strength of S31 (shown in Fig. 1c). The sample breaks when the slope of compressive stress-strain curves turns to be negative. The values of yield compressive strength are 36.4, 15.9 and 6.1 MPa for S11, S21 and S31, respectively.

### Magnetostructural transformation

**Figure 2:**
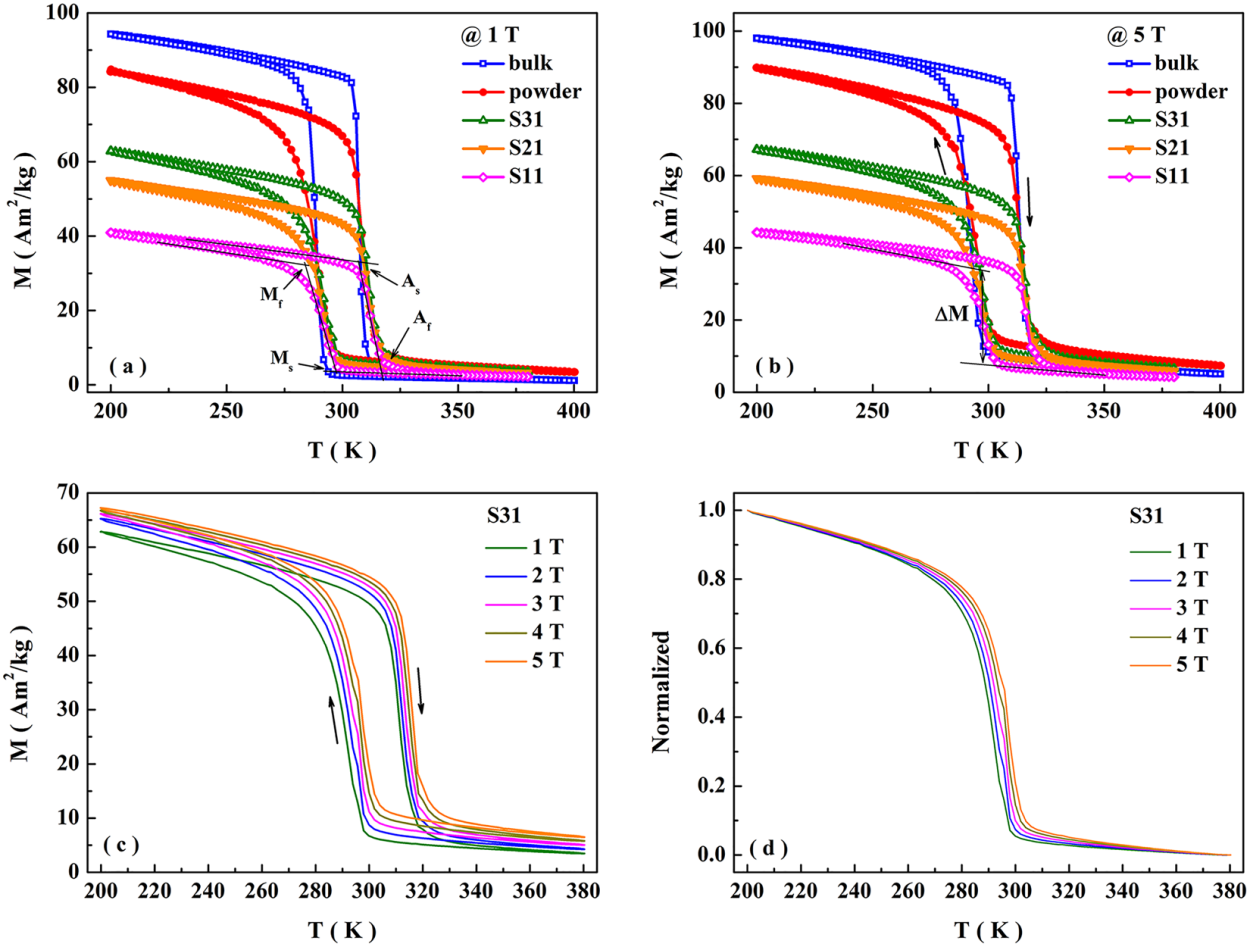
M-T curves for the bulk, powder and composites (S11, S21 and S31) with a magnetic field of 0.1 T (**a**) and 5 T (**b**). The method to obtain M_s_, M_f_, A_s_, A_f_ and ΔM is shown in the curves of S11 as instance. (**c**) M-T curves for S31 with magnetic fields of 0.1, 1, 2, 3, 4 and 5 T. (**d**) Normalized M-T curves for S31 in the cooling process. The black arrow indicates the curves shift to higher temperature by 6.71 K.

The temperature dependence of magnetization (M-T) curves at 1 T (Fig. 2a) indicates the occurrence of first-order magnetostructural transformation between the ferromagnetic O and paramagnetic H phases in all samples. The martensitic start temperature (M_s_), martensitic finish temperature (M_f_), austenitic start temperature (A_s_) and austenitic finish temperature (A_f_) obtained from Fig. 2(a) are listed in Table 1. The magnetostructural transformation in the bulk occurs at around RT, which agrees well with the XRD data. After grinding the bulk into powders, the magnetostructural transformation temperature doesn’t shift obviously, but the transformation width (W) becomes broad. It can be attributed to the so-called “particle size effect”^11^. The transformation widths in the cooling and heating processes are calculated by W(Cooling) = M_s_ − M_f_ and W(Heating) = A_f_ − A_s_, respectively (listed in Table 1). With embedding the powders into the Sn matrix, the values of M_s_, M_f_, A_s_, A_f_ of the composites slightly increase compared with those of the powders, and it can be attributed to the diffusion of Ge atoms into Sn matrix (see Fig. S2 and the corresponding statement in Supplemental information). The M-T curves measured under 5 T (Note that the magnetic field of 5 T is high enough to saturate the ferromagnetic O-phase^12^) are shown in Fig. 2b. Sorting by the saturation magnetization in the low-temperature ferromagnetic phase, the order is the bulk, powders, S31, S21 and S11. As mentioned before, the particle size ranges from 2 to 30 μm after grinding. Due to the so-called size effect^11^, the particles with the size smaller than 5 μm lose the ability of structural transformation and keep at the stable hexagonal phase. Since the Curie-temperature of hexagonal phase is around 200 K^12^, these particles are paramagnetic in the measurement temperature region. Therefore, the magnetization of low-temperature phase in the powder under 5 T is lower than that of the bulk (Fig. 2b). In the composite, the powders are mixed with diamagnetic Sn. In that case, the magnetization of low-temperature phase further decreases with increasing Sn content (Fig. 2b). According to Fig. 2b, the magnetizations of powders, S31, S21 and S11 at 200 K with a magnetic field of 5T are 89.80, 67.23, 59.17 and 44.31 Am^2^/kg. If the diamagnetic response of Sn is ignored, the weight fractions of powders in composites can be calculated as 67.23/89.80 ≈ 74.87%, 59.17/89.80 ≈ 65.89% and 44.31/89.80 ≈ 49.34%, respectively. In this regard, the weight ratios of Mn_0.66_Fe_0.34_Ni_0.66_Fe_0.34_Si_0.66_Ge_0.34_ to Sn are nearly the same with the ingredient. According to the high field M-T curves in Fig. 2b, the ΔM during the transition is calculated (listed in Table 1). The ΔM of S11, S21 and S31 are 28.98, 38.19 and 43.49 Am^2^/kg, which can be converted to 57.96, 57.29 and 57.99 Am^2^/kg if only the magnetization of Mn_0.66_Fe_0.34_Ni_0.66_Fe_0.34_Si_0.66_Ge_0.34_ is considered. These values are almost the same with that of the powders (61.10 Am^2^/kg). Therefore, the Sn-bonding doesn’t obviously reduce the ΔM of Mn_0.66_Fe_0.34_Ni_0.66_Fe_0.34_Si_0.66_Ge_0.34_, and the composites are expected to exhibit an similar rate of the transformation temperature shift by magnetic field as the powders (shown below).

**Table 1:** The martensitic start temperature (M_s_), martensitic finish temperature (M_f_), austenitic start temperature (A_s_), austenitic finish temperature (A_f_), transformation width (W) in heating/cooling process, magnetization difference (ΔM), rate (R) of the temperature shift by magnetic field in the cooling process and entropy change of complete transformation (L) for the bulk, powder and composites.

	M_s_ (K)	M_f_ (K)	A_s_ (K)	A_f_ (K)	W(K) Cooling/Heating	ΔM (Am^2^/kg)	R (K/T)	L (J·kg^−1^·K^−1^)
bulk	291.39	285.03	304.62	310.21	6.36/5.59	77.98	0.77	62.36
powder	295.72	278.69	303.74	313.71	17.03/9.97	61.10	1.61	47.53
S31	297.78	282.93	306.09	316.38	14.85/10.29	43.49	1.36	31.53
S21	296.76	282.36	305.77	316.07	14.40/10.30	38.19	1.37	27.72
S11	297.86	283.38	306.97	316.38	14.48/9.41	28.98	1.43	21.88

According to the Clausius-Clapeyron relation, due to the existence of ΔM between the O and H phases, the magnetostructural transformation in MnMX system can be induced by the magnetic field. The magnetic field-induced magnetostructural transformation manifests as the shift of transformation temperature when applying magnetic field. Taking S31 for instance, the magnetostructural transformation shifts to the higher temperatures with the increase of applied magnetic field, suggesting the magnetic-field-induced magnetostructural transformation (Fig. 2c). This effect can be clearly described when normalizing the curves by

1 $${\rm{v}}=\frac{{\rm{M}}({\rm{T}})-{{\rm{M}}}_{{\rm{H}}}}{{{\rm{M}}}_{{\rm{O}}}-{{\rm{M}}}_{{\rm{H}}}}$$

where M(T) is the magnetization at different temperatures, M_H_ and M_O_ are the magnetizations of O and H phases, respectively. For the simplicity, we use the magnetization at 200 K as M_O_ and the value at 380 K as M_H_. The normalized curves in the cooling process are shown in Fig. 2d. Similar magnetic-field-induced magnetostructural transformation is also found in the bulk, powders and other composites (see Figs S2 and S3 in Supplementary information). The rate (R) of the temperature shift by magnetic field in the cooling process, which is calculated by (M_s_(5T) + M_f_(5T) − M_s_(1T) − M_f_(1T))/2/ΔB(=4T), is shown in Table 1 (M_s_(5T) and M_f_(5T) are listed in Table S1 in Supplementary information). It can be found that the values of R of the powders and composites are larger than that of the bulk.

The magnetic-field-induced magnetostructural transformation can be understood as a process that the grains with H-structure overcome some constraints and transform to O-structure when introducing magnetic field energy. In the bulk, due to the large volume difference between O and H structures, the grains with different structures restrain each other. Therefore, compared with the residual strain and defects, the stress generated from the interfaces between grains is the dominant constraint on the structural transformation^11^. With grinding the bulk into powders, the grains are separated from each other and the interfaces are reduced. So the dominant constraint is largely released in the powders and it becomes much easier to induce the H-O structural transformation by the magnetic field than that in the bulk. But when embedding the powders into the Sn matrix, the occurrence of magnetic-field-induced structural transformation needs to overcome the additional constraint applied by Sn. Therefore, R of the composites is smaller than that of the powders, but is still almost twice as large as that of the bulk.

### Magnetocaloric effect

**Figure 3:**
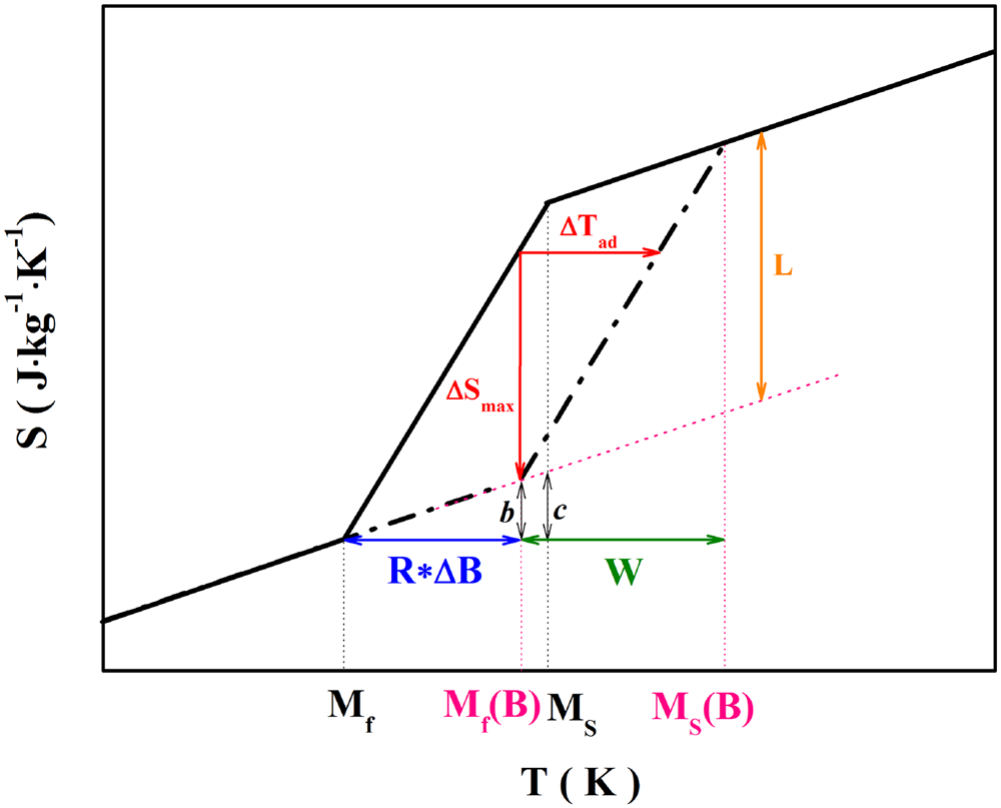
S-T diagram based on the simple model (details in the text).

Accompanied by the magnetic-field-induced structural transformation, the magnetocaloric effect can be obtained. In this work, the magnetocaloric effect is estimated by a simple model based on the entropy-temperature (S-T) diagram, which is also mentioned in ref.^35^. In this model, the S-T diagram is built by drawing the tangent lines at the inflection points of the two entropy curves in zero field and an applied magnetic field, thus the area in the diagram is a parallelogram. As shown in Fig. 3, the black solid and dotted lines represent the temperature-dependent entropy under zero field and applied magnetic field, respectively. M_s_(B) and M_f_(B) are the martensitic start and finish temperatures under a magnetic field of B. The entropy change of complete transformation (L) (also called the latent heat) is determined from the DSC data (shown in Fig. S1) by

2 $${\rm{L}}={\int }_{{{\rm{M}}}_{{\rm{f}}}}^{{{\rm{M}}}_{{\rm{s}}}}\frac{(\dot{{\rm{Q}}}-{\dot{{\rm{Q}}}}_{{\rm{baseline}}})}{{\rm{T}}}{(\frac{\partial {\rm{T}}}{\partial {\rm{t}}})}^{-1}{\rm{dT}}$$

where $\dot{{\rm{Q}}}$ is the heat flow per mass unit and $\dot{{\rm{Q}}}$_baseline_ can be obtained by adjusting a smooth line at temperatures below and above the transition anomalies^36^. The calculated L of the bulk, powders and composites are also listed in Table 1. The isothermal entropy change (ΔS) and adiabatic temperature change (ΔT_ad_) can be obtained by measuring the length of the perpendicular and horizontal arrows indicated in Fig. 3. According to the geometrical proportions, it can be found that the maximum ΔS (ΔS_max_) will appear in the temperature range between M_f_(B) and M_s_. The ΔS_max_ and ΔT_ad_ can be linked by

3 $$\frac{{\rm{R}}\cdot {\rm{\Delta }}{\rm{B}}}{{{\rm{\Delta }}{\rm{T}}}_{{\rm{ad}}}}=\frac{{{\rm{\Delta }}{\rm{S}}}_{{\rm{\max }}}+b}{{{\rm{\Delta }}{\rm{S}}}_{{\rm{\max }}}}$$

4 $$\frac{{\rm{W}}}{{{\rm{\Delta }}{\rm{T}}}_{{\rm{ad}}}}=\frac{{\rm{L}}+c}{{{\rm{\Delta }}{\rm{S}}}_{{\rm{\max }}}}$$

Different from ref.^35^, we bring

5 $$\frac{b}{c}=\frac{{\rm{R}}\cdot {\rm{\Delta }}{\rm{B}}}{{\rm{W}}}$$

into Eqs 3 and 4, and the ΔS_max_ can be expressed as

6 $${{\rm{\Delta }}{\rm{S}}}_{{\rm{\max }}}=\frac{{\rm{L}}\cdot {\rm{R}}\cdot {\rm{\Delta }}{\rm{B}}}{{\rm{W}}}$$

So the obtained ΔS_max_ is determined by the latent heat, the rate of transformation temperature shift by magnetic field and transformation width. When W is lower than the shift of entropy curves under the applied magnetic field, the ΔS_max_ should be just equal to L, because the magnetic field can induce a complete transformation in this temperature region. Based on this, the ΔS_max_ should be written as following:

7 $${{\rm{\Delta }}{\rm{S}}}_{{\rm{\max }}}=\{\begin{array}{ll}{\rm{L}} & ({\rm{W}} < R\cdot {\rm{\Delta }}B)\\ \frac{{\rm{L}}\cdot {\rm{R}}\cdot {\rm{\Delta }}{\rm{B}}}{{\rm{W}}} & ({\rm{W}} > R\cdot {\rm{\Delta }}B)\end{array}$$

**Figure 4:**
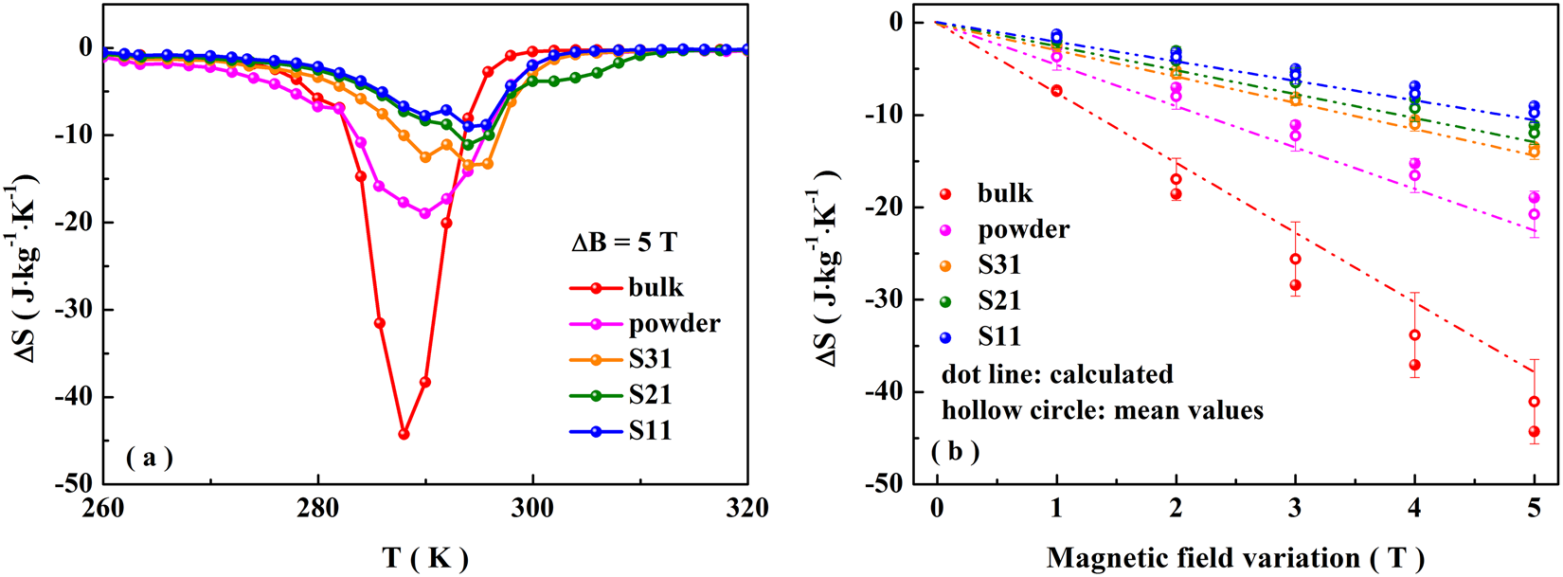
(**a**) Isothermal entropy change for the bulk, powder and composites with the magnetic field variation of 5 T. (**b**) Magnetic field variation dependence of ΔS_max_ obtained by Maxwell relation (circle) and Eq. 6 (dotted line). The calculated mean values is indexed as hollow circle.

In the condition of ΔB = 5 T, W is still larger than R·ΔB for all our composite samples. Therefore, the values of ΔS_max_(0–5 T) can be calculated by Eq. 6 using the data listed in Table 1, and they are −37.69, −22.40, −14.44, −13.16 and −10.76 J·kg^−1^·K^−1^ for the bulk, powder, S31, S21 and S11, respectively. According to the M-T curves under different magnetic fields, the Maxwell relation is also used to confirm the ΔS_max_ (0–5 T). As shown in Fig. 4a, the corresponding values are −44.28, −18.97, −13.43, −11.10 and −9.02 J·kg^−1^·K^−1^, which are in accordance with that calculated by Eq. 6. Based on the model and experimental data, the mean values and standard deviation are calculated (shown in the Fig. 4b). It also indicates a good accordance. Sorting by the largest magnetic entropy change, the order is the bulk, powder, S31, S21 and S11. After grinding the bulk into powder, W increases (see Table 1). It leads to the reduced ΔS_max_ because there is an inverse relationship between ΔS_max_ and W. On the other hand, the fraction of particles with stable phase causes a decrease of L in per unit mass, which also leads to the reduced ΔS_max_. Although R is increased, it is not high enough to prevent the decrease of ΔS_max_. With embedding the powder into Sn, R and W don’t change obviously (see Table 1), but the L is reduced because of the existence of Sn. Therefore, ΔS_max_ further decreases as Sn-content increases. Although ΔS_max_ is reduced, the composites exhibit an improved machinability. On the other hand, the values of full width at half maximum for S31, S21 and S11 are 12.72, 11.43 and 12.55 K, which are larger than that of the bulk (7 K), indicating the broadened operating temperature range.

Additionally, the observed thermal hysteresis in composite is higher than 20 K and the shift of transformation temperature in a reasonable magnetic field is relatively lower than that in the other magnetic-transition alloys, such as La(Fe,Si)_13_ and MnFe(P,Si) alloys^25–27,31,37^. In that case, the magnetic-field-excited orthorhombic phase maintains after the field is removed, and no metamagnetic transition occurs during the second field cycle. Thus, the magnetocaloric effect is nearly zero in cycling condition. The irreversibility of magnetocaloric effect hinders its application as magnetic cooling refrigerant. To enhance the reversibility, how to greatly reduce the thermal hysteresis and increase the rate of the transformation temperature shift by magnetic field is the question that is worth thinking about.

## Conclusions

In summary, the Mn_0.66_Fe_0.34_Ni_0.66_Fe_0.34_Si_0.66_Ge_0.34_/Sn composites are prepared by hot pressing. The magnetostructural transformation and magnetocaloric effect of these composites are investigated in this work. The Mn_0.66_Fe_0.34_Ni_0.66_Fe_0.34_Si_0.66_Ge_0.34_/Sn composites display the magnetostructural transformation between ferromagnetic O and paramagnetic H phases. Due to the reduced size of Mn_0.66_Fe_0.34_Ni_0.66_Fe_0.34_Si_0.66_Ge_0.34_ grains, the composites exhibit a broadened magnetostrutural transformation and an improved rate of the transformation temperature shift by magnetic field relative to those of the bulk. Accompanied by the occurrence of magnetic-field-induced magnetostructural transformation, these composites exhibit magnetocaloric effect. The isothermal entropy change is calculated by a simple model based on S-T diagram. The obtained results are consistent with that obtained by Maxwell relation.

## Methods

The Mn_0.66_Fe_0.34_Ni_0.66_Fe_0.34_Si_0.66_Ge_0.34_ precursor was prepared by the method mentioned in ref.^12^. For synthesizing the Mn_0.66_Fe_0.34_Ni_0.66_Fe_0.34_Si_0.66_Ge_0.34_/Sn composite, the precursor alloy was ground into powders using a ceramic mortar by hand, and then mixed with the commercial Sn powders with an average size of 43 μm for one more hour grinding using an agate mortar. The mixed powders were hot-pressed at 280 °C under 250 MPa for 5 min in vacuum and then slowly cooled to RT in 6 hrs. The applied pressure is maintained till the sample is cooled to RT.

The structural transition was investigated by DSC (Mettler Toledo, DSC 3) with a ramp rate of 10 K/min. The structural characterization was performed by XRD (Bruker, D8 Advance) at RT with Cu-Ka radiation. The cross-sectional microstructure was observed by SEM (FEI Quanta 250F). The elemental mapping image was obtained by energy-dispersive spectroscopy (FEI Quanta 250F). The M-T curve was carried out using a Physical Property Measurement System (Quantum Design, Dynacool) with a ramp rate of 2 K/min. The yield compressive strength was tested by an universal testing machine. The thermal expansion was investigated by thermomechanical analysis (402 F3 Hyperion).

### Data availability

All relevant data are available from authors upon reasonable request.

## Electronic supplementary material

supplementary information
