# Supplementary material for: Magnetostructural transformation and magnetocaloric effect of Sn-bonded Mn0.66Fe0.34Ni0.66Fe0.34Si0.66Ge0.34 composite
Source: Sci Rep. 2018 Jan 8;8:19. doi: 10.1038/s41598-017-18240-x (PMC5758529; doi:10.1038/s41598-017-18240-x)
Supplement: Supplementary file 1 — supplementary information [file 41598_2017_18240_MOESM1_ESM.pdf]

Supplementary information for "Magnetostructural transformation and magnetocaloric effect of Sn-bonded $Mn_{0.66}Fe_{0.34}Ni_{0.66}Fe_{0.34}Si_{0.66}Ge_{0.34}$ composite"

Yu Si, Jun Liu, Yuan-yuan Gong*, Sheng-yun Yuan, Guo Peng, Gui-zhou Xu, Feng Xu**

School of Materials Science and Engineering & Herbert Gleiter Institute of Nanoscience, Nanjing University of Science and Technology, Nanjing 210094, China

* Corresponding author. E-mail: gyy@njust.edu.cn, Tel: +86-25-84303411.

** Corresponding author. E-mail: xufeng@njust.edu.cn, Tel: +86-25-84303411.

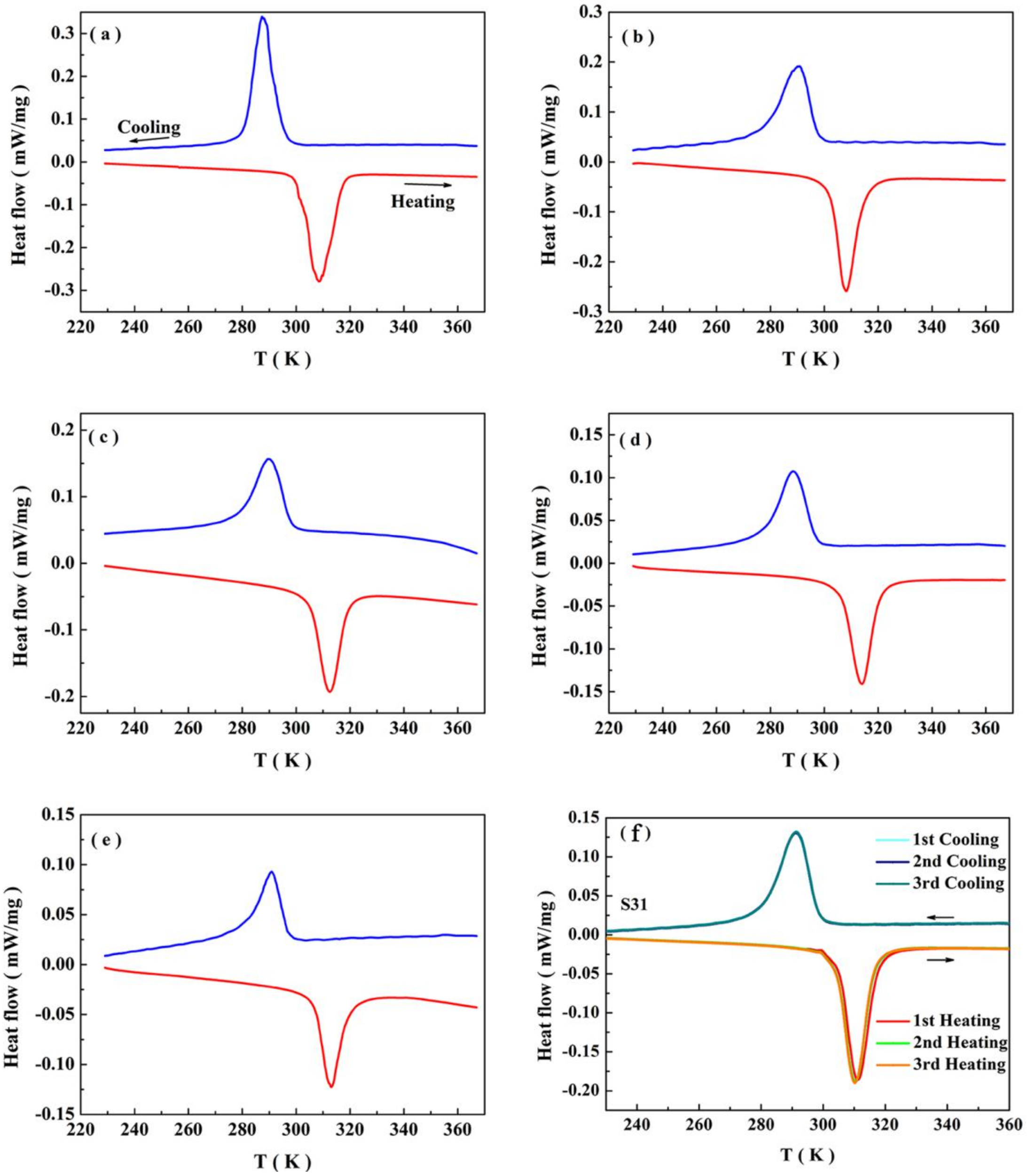

Fig. S1. DSC curves for the bulk **(a)**, powder **(b)**, S31 **(c)**, S21 **(d)** and S11 **(e)**. Compilations of three DSC cycles for S31 **(f)**.

The endothermic/exothermic curves of S31 almost repeat each other in three cycles (Fig. S1(f)), and the transformation temperature doesn't obviously shift after repeated heating/cooling cycles. Similar behaviors were also observed in S11 and S21 (not shown here).

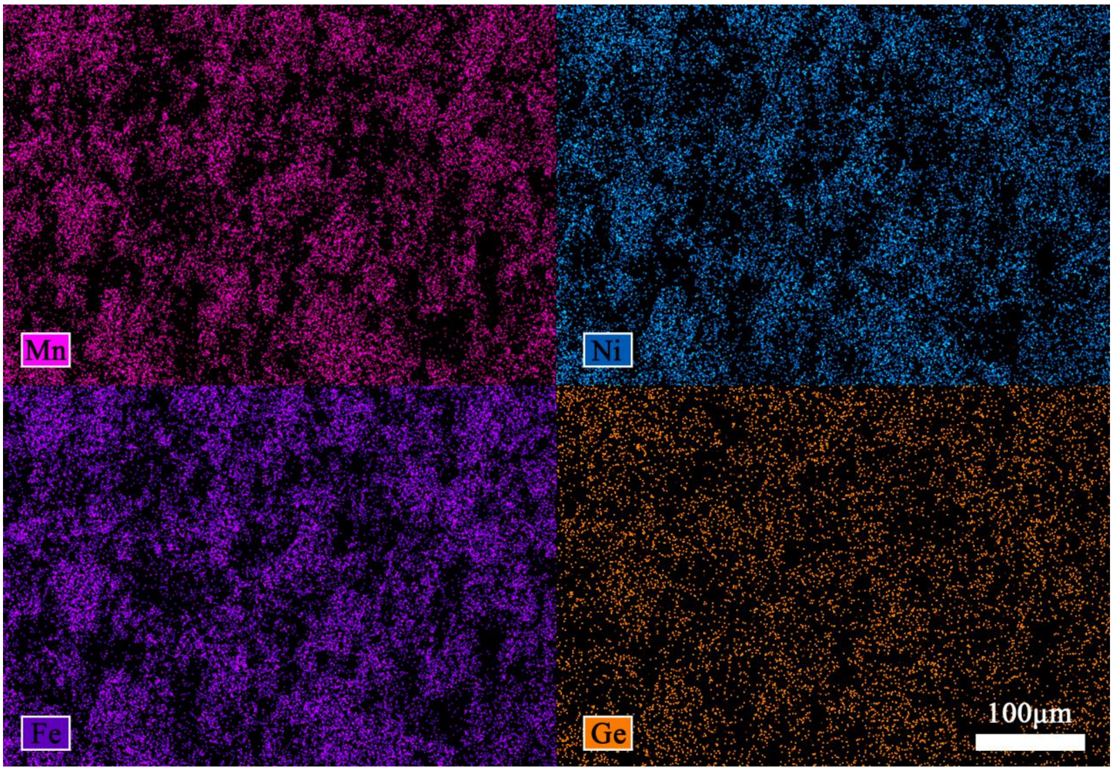

Fig. S2. The elemental mapping images of Mn, Ni, Fe and Ge for S11.

By comparing the elemental mapping of Mn, Ni, Fe and Ge, it can be found that a small amount of Ge atoms diffuse into Sn matrix during hot-pressing. It is known that the structural transformation temperature is sensitive to the content of main-group element. With the decrease of Ge-content, the structural transformation temperature will increase.

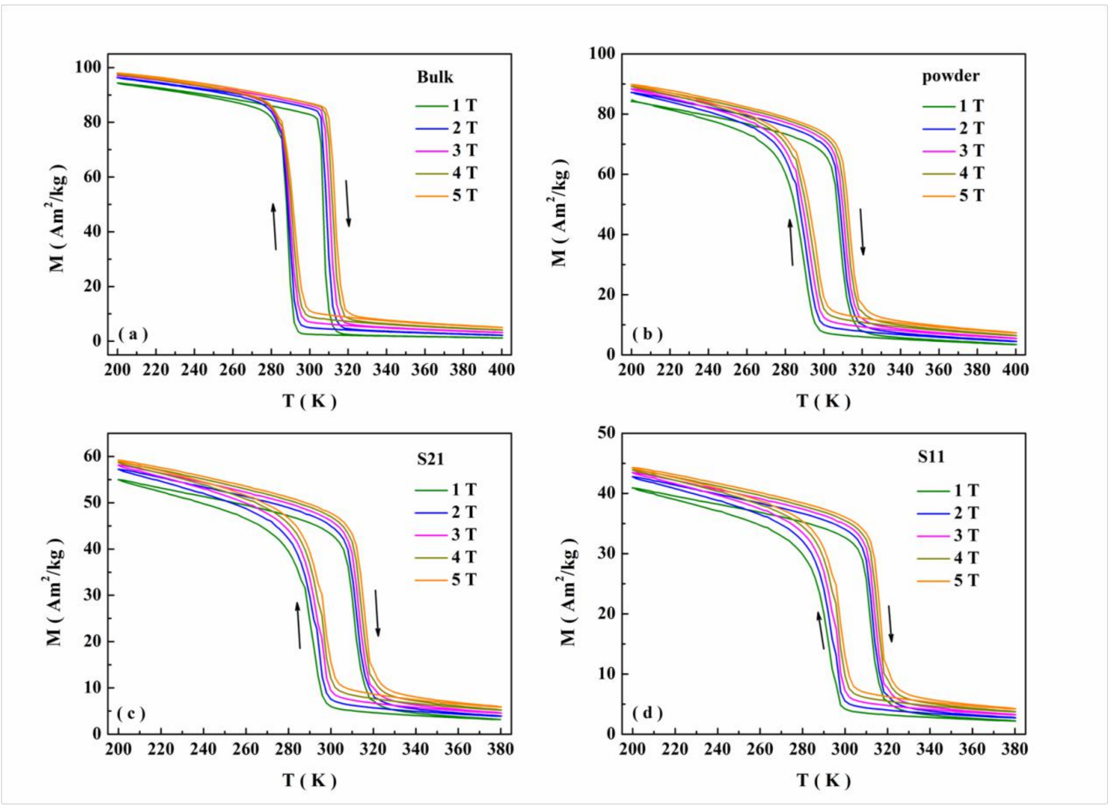

Fig. S3. M-T curves for bulk **(a)**, powder **(b)**, S21 **(c)** and S11 **(d)** with magnetic fields of 1, 2, 3, 4 and 5 T.

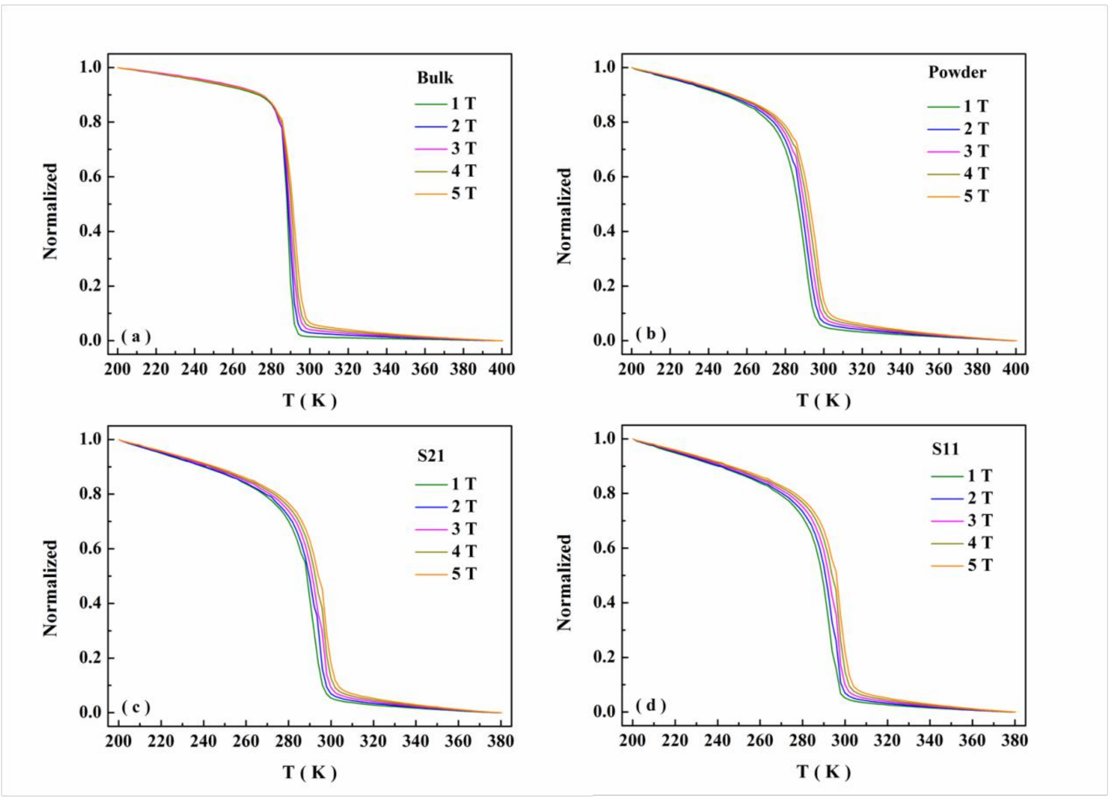

Fig. S4. Normalized M-T curves for bulk **(a)**, powder **(b)**, S21 **(c)** and S11 **(d)** in the cooling process.

Table S1.

The martensitic start temperature (M_s) and martensitic finish temperature (M_f) for the bulk, powder and composites with an applied magnetic field of 5 T.

	**Bulk**	**Powder**	**S31**	**S21**	**S11**
**M_s (K)**	297.03	301.51	303.06	302.27	303.56
**M_f (K)**	285.54	285.74	288.53	287.79	289.08
